# Supplementary material for: Idiosyncratic Fitness Costs of Ampicillin-Resistant Mutants Derived from a Long-Term Experiment with Escherichia coli
Source: Antibiotics (Basel). 2022 Mar 6;11(3):347. doi: 10.3390/antibiotics11030347 (PMC8944548; doi:10.3390/antibiotics11030347)
Supplement: Supplementary file 1 [file antibiotics-11-00347-s001.zip › antibiotics-1607443-supplementary.pdf]

Supplemantary Material

Table S1. Bacterial strains used in this study.

**Evolved ampicillin-resistant clones**		
**Strain name**	**Derived from**	**Freezer ID**
Ancestor-1	REL606	KJC108
Ancestor-2	REL606	KJC109
Ancestor-3	REL606	KJC110
Ancestor-4	REL606	KJC111
Ara–5-1	REL11339	KJC114
Ara–5-2	REL11339	KJC122
Ara–5-3	REL11339	KJC130
Ara–6-1	REL11389	KJC115
Ara–6-2	REL11389	KJC123
Ara–6-3	REL11389	KJC131
Ara+4-1	REL11348	KJC112
Ara+4-2	REL11348	KJC120
Ara+4-3	REL11348	KJC128
Ara+5-1	REL11367	KJC113
Ara+5-2	REL11367	KJC121
Ara+5-3	REL11367	KJC129
**Ampicillin-sensitive parental strains**		
**LTEE population**	**LTEE generation**	**Freezer ID**
Ancestor	0	REL606
Ara–5	50,000	REL11339
Ara–6	50,000	REL11389
Ara+4	50,000	REL11348
Ara+5	50,000	REL11367
**Strains used as common competitors**		
**LTEE population**	**LTEE generation**	**Freezer ID**
Ancestor	0	REL607
Ara–5	40,000	REL10948
Ara–5	40,000	REL11638
